# Combination of oncolytic Maraba virus with immune checkpoint blockade overcomes therapy resistance in an immunologically cold model of advanced melanoma with dysfunctional T-cell receptor signalling

**DOI:** 10.1136/jitc-2024-009443

**Published:** 2024-07-25

**Authors:** Edward Armstrong, Matthew K L Chiu, Shane Foo, Lizzie Appleton, Pablo Nenclares, Anton Patrikeev, Nitya Mohan, Martin Mclaughlin, Galabina Bozhanova, Julia Hoebart, Victoria Roulstone, Emmanuel Patin, Malin Pedersen, Joan Kyula, Masahiro Ono, Fiona Errington-Mais, John Bell, Kevin J Harrington, Alan Melcher, Victoria Jennings

**Affiliations:** 1 Division of Radiotherapy and Imaging, The Institute of Cancer Research, London, UK; 2 Department of Clinical Oncology, University of Hong Kong Faculty of Medicine, Hong Kong, Hong Kong; 3 Imperial College London, London, London, UK; 4 The Institute of Cancer Research, London, UK; 5 Head and Neck Unit, Royal Marsden Hospital NHS Trust, London, UK; 6 Leeds Institute of Medical Research at St. James’s, University of Leeds, Leeds, UK; 7 Cancer Therapeutics, Ottawa Hospital Research Institute, Ottawa, Ontario, Canada; 8 Leeds Institute of Medical Research, University of Leeds, Leeds, UK

**Keywords:** Oncolytic virus, Immune Checkpoint Inhibitor, T cell, Immunotherapy, Immunosuppression

## Abstract

**Background:**

Over the past decade, cancer immunotherapies have revolutionized the treatment of melanoma; however, responses vary across patient populations. Recently, baseline tumor size has been identified as an independent prognostic factor for overall survival in patients with melanoma receiving immune checkpoint inhibitors. MG1 is a novel oncolytic agent with broad tumor tropism that has recently entered early-phase clinical trials. The aim of this study was to characterize T-cell responses in human and mouse melanoma models following MG1 treatment and to establish if features of the tumor immune microenvironment (TIME) at two distinct tumor burdens would impact the efficacy of oncolytic virotherapy.

**Methods:**

Human three-dimensional in vitro priming assays were performed to measure antitumor and antiviral T-cell responses following MG1 infection. T-cell receptor (TCR) sequencing, T2 killing assay, and peptide recall assays were used to assess the evolution of the TCR repertoire, and measure specific T-cell responses, respectively. In vivo, subcutaneous 4434 melanomas were characterized using RNA sequencing, immunohistochemistry, and flow cytometry. The effectiveness of intratumoral MG1 was assessed in advancing 4434 tumors and the generation of antitumor and antiviral T cells measured by splenocyte recall assays. Finally, combination MG1 and programmed cell death protein-1 antibody (αPD-1) therapy was investigated in advanced 4434 tumors.

**Results:**

MG1 effectively supported priming of functional cytotoxic T cells (CTLs) against tumor-associated antigens as well as virus-derived peptides, as assessed using peptide recall and T2 killing assays, respectively. TCR sequencing revealed that MG1-primed CTL comprised larger clusters of similar CDR3 amino acid sequences compared with controls. In vivo testing of MG1 demonstrated that MG1 monotherapy was highly effective at treating early disease, resulting in 90% cures; however, the efficacy of MG1 reduced as the disease burden (local tumor size) increased, and the addition of αPD-1 was required to overcome resistance in more advanced disease. Differential gene expression profiles revealed that increased tumor burden was associated with an immunologically colder TIME. Furthermore, analysis of TCR signaling in advancing tumors demonstrated a different dynamic of TCR engagement compared with smaller tumors, in particular a shift in antigen recognition by CD4+ cells, from conventional to regulatory subsets.

**Conclusion:**

Addition of αPD-1 to MG1 is required to overcome viral therapy resistance in immunologically ‘colder’ more advanced melanoma, highlighting the importance of tumor burden to different types of immunotherapy.

**WHAT IS ALREADY KNOWN ON THIS TOPIC**

- Oncolytic viruses are promising immunotherapeutic agents, but their efficacy, particularly following intratumoral injection, in relation to the size of the targeted tumor, is unclear.

**WHAT THIS STUDY ADDS**

- Here we show that an oncolytic rhabdovirus, Maraba, activates an immune response in human as well as mouse preclinical systems, which targets viral as well as tumor-associated antigens. The benefit of immune checkpoint inhibitor (ICI) to virotherapy is apparent only on the treatment of larger tumors, which have an inherently more immunosuppressive tumor microenvironment, including skewing of T-cell receptor engagement with antigen from conventional to regulatory CD4+T cells. Maraba converts the immunologically desert tumor to immune inflamed, thus priming the tumor microenvironment for effective programmed cell death protein-1 antibody (αPD-1) combination therapy.

**HOW THIS STUDY MIGHT AFFECT RESEARCH, PRACTICE OR POLICY**

- This study highlights the impact of tumor burden on the immune microenvironment and demonstrates how this impacts effective oncolytic virotherapy. Finally, it supports the use of Maraba oncolytic virotherapy in combination with ICIs in advance melanoma.

## Introduction

 Oncolytic viruses (OVs) preferentially replicate within cancer cells, causing direct cytotoxicity, and inducing both innate and adaptive antitumor immune responses. To date, a multitude of OVs have been tested in clinical trials and have been safe and well tolerated. The most clinically advanced agent, and the only virus currently approved for clinical use across the USA, Europe, and Australasia, for local treatment of unresectable metastatic melanoma, is a genetically modified double-stranded DNA herpes simplex virus (JS-1 strain), talimogene laherparepvec (T-Vec; IMLYGIC, Amgen). The generation of local and systemic immune responses following oncolytic virotherapy has supported the rationale to combine OVs with other cancer immunotherapies, such as immune checkpoint inhibitors (ICIs). The MASTERKEY-265 (ClinicalTrials.gov: NCT02263508) trial, evaluated T-Vec in combination with pembrolizumab, a programmed cell death protein-1 antibody (αPD-1), for patients with advanced melanoma (stage IIIB–IVM1c). The Phase Ib part of this trial recruited 21 patients and confirmed that treatment was well tolerated, with no dose-limiting toxicities and encouraging early efficacy signals.^1^ However, the full randomized, double blind, Phase III study (ClinicalTrials.gov: NCT02263508) was stopped early due to clinical futility, perhaps because the outcome in the patient population tested was too good with pembrolizumab monotherapy for the addition of OV to make a significant difference, and/or because the protocol was altered between the early and later stages of the trial.^2^

Maraba virus was first developed as an oncolytic agent in 2010, and is a single-stranded, negative-sense, enveloped RNA virus that derives from the vesiculovirus genus of the Rhabdoviridae. Genetic modifications to the wild-type virus have resulted in the development of MG1, which has an enhanced capacity to replicate within tumor cells, a superior propensity to induce cancer cell death^3^ and has recently entered early clinical testing.^4 5^ MG1 has shown both oncotropic and cytotoxic activity in a range of murine and human cell lines. Preclinical studies have shown the successful application of MG1 as (1) a monotherapy,^6^ (2) a cancer vaccine vector expressing either tumor-associated or viral antigens,^7^,^9^ (3) in combination with standard of care chemotherapeutic agents^10^ and (4) in combination with ICI in a neoadjuvant setting.^11^ All these features support the potential use of MG1 as an immunogenic oncolytic viral therapeutic agent. Despite a significant amount of preclinical data supporting MG1 as a potent oncolytic agent capable of generating antitumor immunity in murine cancer models, to date there is limited data on the ability of MG1 to support the generation of human antitumor T-cell responses, and what impact tumor size has on antitumor immune activation by MG1 or indeed other OVs. Therefore, the aim of this study was to monitor the generation and magnitude of antitumor and antiviral T-cell responses following MG1 treatment in both human and murine melanoma preclinical models. To do this we first used a human in vitro three-dimensional (3D) melanoma T-cell priming assays and measured primed cytotoxic T cells (CTL) responses against melanoma-associated antigens, and against specific human leukocyte antigen- A2 (HLA-A2)-restricted viral peptides. In addition, we performed T-cell receptor (TCR) sequencing to track the evolution of the human TCR repertoire during MG1-induced CTL priming assays. In mice, using an early-stage and late-stage disease model of melanoma, we demonstrated that tumor size impacts on the tumor immune microenvironment (TIME), consistent with other data with differing disease burdens,^12 13^ and that a further feature of dysfunctional immunity in larger tumors is differences in CD4+TCR engagement with antigen. MG1 monotherapy activated the TIME and was effective against small tumors generating long-lasting antitumor immunity. For advance disease, however, the addition of αPD-1 therapy to MG1 was required to result in a significant survival benefit.

## Results

### MG1 infects, replicates in, and is cytotoxic against, melanoma cell lines grown either as 2D or 3D cultures

**Figure 1:**
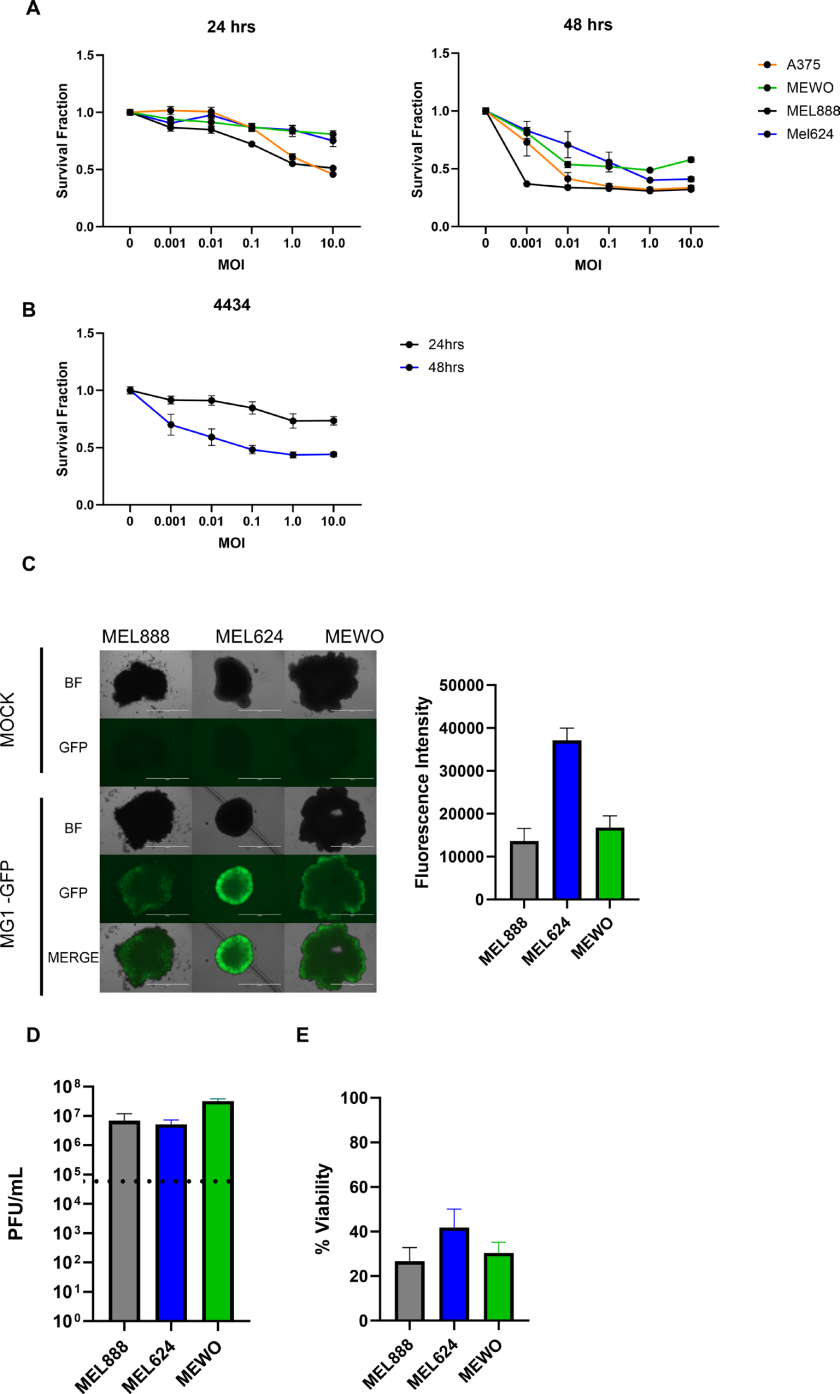
MG-1 infects, replicates and is cytotoxic against melanoma cell lines grown either in 2D or 3D cultures. Human (A) and murine (B) melanoma cell lines were treated with MG-GFP at concentrations ranging from 0 to 1 PFU/cell, cell viability was determined by MTT assay at 24 and 48 hours. Data shown is the average of three independent experiments±SEM. (C/D and E) Human melanoma tumor spheroids were infected with MG-GFP at an MOI 0.1, bright field and fluorescence images were taken and quantified at 20 hours (C). Viral replication was determined at 24 hours, input doses indicated with dotted line (D) and viability measured at 48 hours (E). Data shown is the average of three independent experiments+SEM. MG1-GFP, MG-1 expressing green fluorescent protein; 2D, two-dimensional; 3D, three-dimensional.

Previous studies have tested MG1 oncolytic activity against five human melanoma cell lines from the NCI60 (US National Cancer Institute) including M14, MALME3M, SKMEL28, UACC257 and UACC6 and three murine melanoma cell lines, B16, B16F10 and B16lacZ.^3 7 14^ To expand on this work, we investigated the oncolytic activity of MG1 in further four human melanoma cell lines (A375, MEWO, MEL888 and MEL624) and the 4434 murine melanoma cell line. A dose and time dependent cytotoxicity was observed in all four human cell lines (figure 1A) and in the murine 4434 cell line (figure 1B) when grown as two-dimensional (2D) monolayers. However, as 3D cultures better mimic the physical and biochemical features of a solid tumor mass, the ability of MG1 to infect, replicate in, and kill human melanoma cells grown in 3D cultures was next investigated. MEL888, MEWO and MEL624 cells all grew as 3D spheroid cultures and were successfully infected with MG-1 expressing green fluorescent protein (MG1-GFP) (figure 1C). Viral replication (figure 1D) and cytotoxicity (figure 1E) were observed in all three cell lines tested. Hence, MG1 retains its oncolytic activity in tumor cells grown in 3D cultures.

### MG1 primes specific antitumor and antiviral T-cell responses

**Figure 2:**
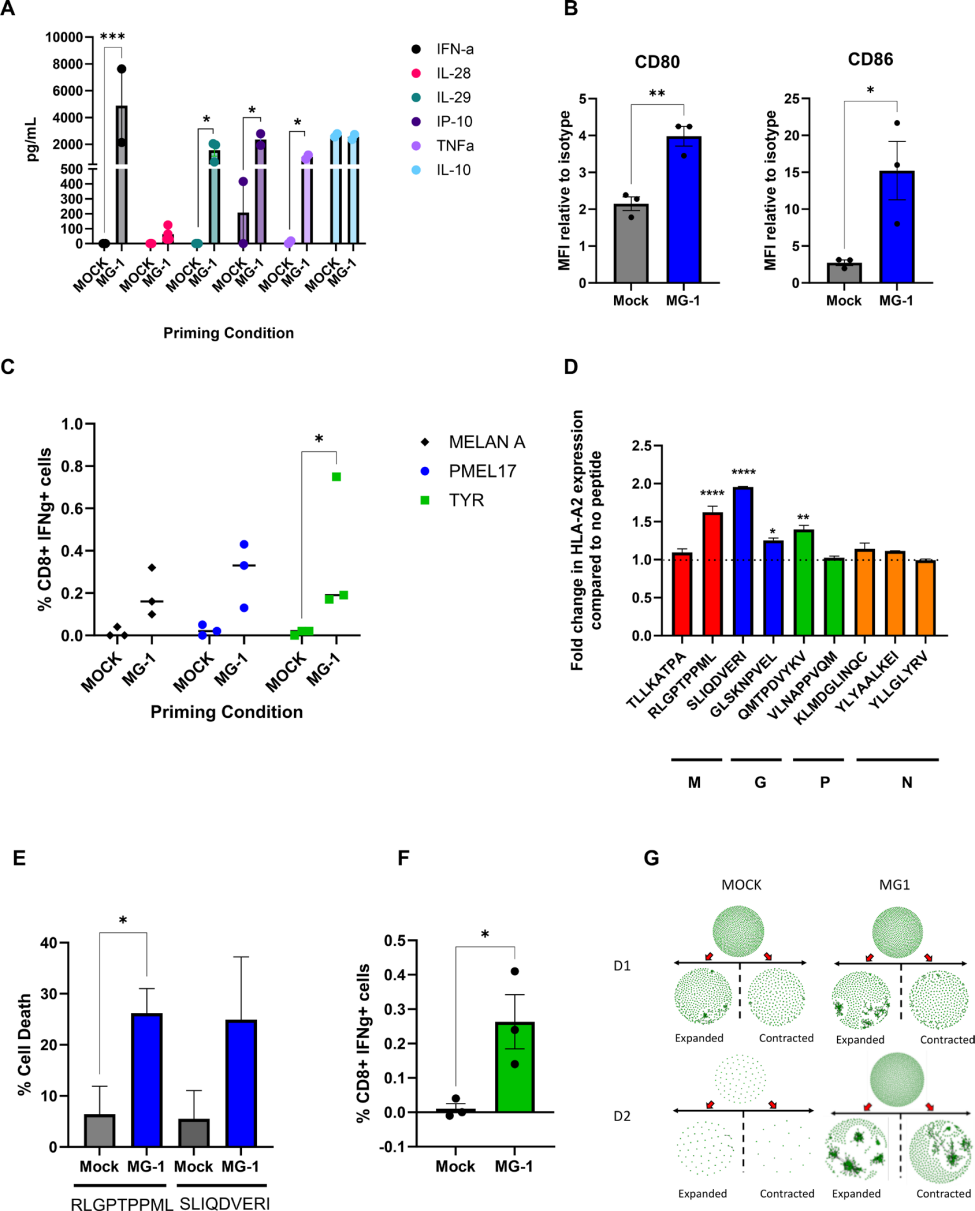
MG1 primes specific antitumor T-cell responses. (A) Supernatants from MEL888 spheroids cells treated with ±MG-1 and cultured with immature dendritic cells (iDC) for 24 hours were collected and concentrations of IFNα, IL-28, IL-29, IP-10, TNFα and IL-10 were determined by ELISA. Data shows the mean+SEM from three independent experiments. (B) iDC were treated with ±MG1 for 48 hours, cell surface expression of CD80 and CD86 was determined by flow cytometry. The mean fold increase in expression compared with isotype controls+SEM are shown (n=3). (C) MEL888 spheroids were treated with ±MG1 and cultured with iDC for 24 hours before single-cell suspensions were removed and cultured with autologous PBMC. CTLs were re-stimulated once more then used in peptide recall assays against melanoma TAAs. The mean percentage CD8 cells expressing IFNγ minus control CD14 stimulation from three donors are shown. (D) T2 cells were incubated with indicated MG1 peptides then cell surface expression of HLA-A2 was determined by flow cytometry, mean fold change in expression compared with no peptide control+SEM are shown (n=2). (E and F) MEL624 spheroids were treated ±MG1 and CTL generated as described in (C). (E) 4-hour Cr^51^ killing assay against T2 cells loaded with indicated peptides. (F) Peptide recall assay against TYR, mean percentage of CD8 cell expressing IFNγ from three donors are shown. (G) Network diagrams of CDR3β amino acid clustering at week-1 (top) and week-2 expanded and contracted repertoires based on normalized expansion rate for each donor (D1 and D2). Each circle within the network represents a TCR clone and each cluster is represented by a group of TCRs linked by vectors. CTLs, cytotoxic T cell; HLA-A2, Human leukocyte antigen A2; IL, Interleukin; IP, Interferon-gamma stimulated protein; MELAN, melanoma antigen recognized by T-cells 1; MFI, median fluorescence Intensity; PBMC, peripheral blood mononuclear cells;TAAs, tumor-associated antigens; TCR, T-cell receptor; TNF, tumour necrosis factor; TYR, tyrosinase.

We have previously developed in vitro, preclinical assays, to test the potential of OVs to support the activation of human adaptive antitumor immune priming using infected 2D monolayers as the melanoma “antigen source”, loaded on to immature dendritic cells (iDC) as antigen-presenting cells (APC), for subsequent co-culture with responder T cells, to generate CTLs.^15^ To increase clinical relevance, we adapted our model system to better mimic human disease and developed a 3D in vitro immune priming assay to test OV-induced antitumor immune priming.^16^ To use 3D tumors as the “antigen load”, MEL888 spheroids were first infected with MG1 and cultured with iDC for 24 hours; cell-free supernatants were then collected and assessed for a range of antiviral, pro-inflammatory and immunosuppressive cytokines. MG1 infection significantly induced the production of interferon (IFN)-α, interleukin (IL)-29, tumour necrosis factor (TNF)-α and interferon gamma-induced protein (IP)-10, while a small increase in IL-28 was detected following MG-1 this was not significant, the levels of the immunosuppressive cytokine IL-10 remained unaltered (figure 2A). As DC maturation is critical for effective T-cell priming, the ability of MG1 to directly induce iDC maturation was next investigated. iDCs cultured with MG1 significantly increased cell surface expression of co-stimulatory molecules CD80 and CD86 (figure 2B). To assess whether this MG1-induced DC phenotype, and pro-inflammatory cytokine production, supported adaptive immune priming, we tested MG1 infected MEL888 spheroids in CTL priming assays and showed that virus infection increased the production of melanoma-specific tumor-associated antigen (TAA) T-cell responses (figure 2C). In addition, since anti-TAA priming has not previously been compared with anti-OV human T-cell priming, we also investigated the generation of antiviral responses under the same CTL priming conditions. NetMHCPan V.4.1 was used to predicted the most immunogenic HLA-A2-restricted peptides in the MG1 proteins M, G, N and P. T2 binding assays were then performed to confirm the ability of these 8-9mers to bind HLA-A2; the M protein peptide, RLGPTPPML, and the G protein peptide, SLIQDVERI, demonstrated the greatest significance in their ability to stabilize HLA-A2 on the surface of T2 cells (figure 2D), indicating strong binding of these two peptides to the HLA-A2 molecule. To test the generation of antiviral CTLs targeting these HLA-A2 restricted virus peptides, MEL624 (HLA-A2+) spheroids, with or without MG1 infection, were loaded onto HLA-A2+iDC, and CTL priming assays were performed, as for the generation of anti-TAA responses. Primed CTL demonstrated increased cytotoxicity against RLGPTPPML and SLIQDVERI peptide-loaded T2 cells compared with unloaded controls, reaching significance for RLGPTPPML (figure 2E). Peptide recall responses against tyrosinase (TYR) overlapping peptide pools were also performed to confirm effective antitumor immune priming alongside antiviral T-cell responses (figure 2F). Finally, TCR sequencing was performed on these primed CTL to explore the evolution of the TCR repertoire during T-cell priming. TCR sequences were tracked between week 1 (one stimulation) to week 2 (two stimulations) from two donors (D1 and D2), to determine whether individual T-cell clones were expanding or contracting. TCRs were clustered based on CDR3 amino acid triplet similarity using a kernel matrix. Week-2 clonotypes were then classified according to their normalized expansion rate ((frequency at week-2/singleton frequency at week-2)/ (frequency at week-1)/singleton frequency at week-1) as expanded (if normalized expansion rate>1) or contracted (if normalized expansion rate<1). Expansion and contraction of TCR clonotypes could be detected in both MG1-primed and mock-primed CTL. Interestingly however, clustering analysis of expanded clonotypes revealed that MG1-primed CTL comprised larger clusters of similar TCR sequences compared with the mock samples (figure 2G, average cluster size in expanded clonotypes=21.5 vs 4.1), potentially representing the development of larger clusters of related T-cell clonotypes targeting the virus and/or TAA specific antigens. Taken together, these data show for the first time that MG1 can support priming of human antitumor T-cell responses, that the TCR repertoire of CTL during priming can be tracked and characterized, and that the functionality of the primed CTL can be measured using virus as well as TAA peptide recall assays.

### Advanced 4434 tumors are immunologically colder than early disease

**Figure 3:**
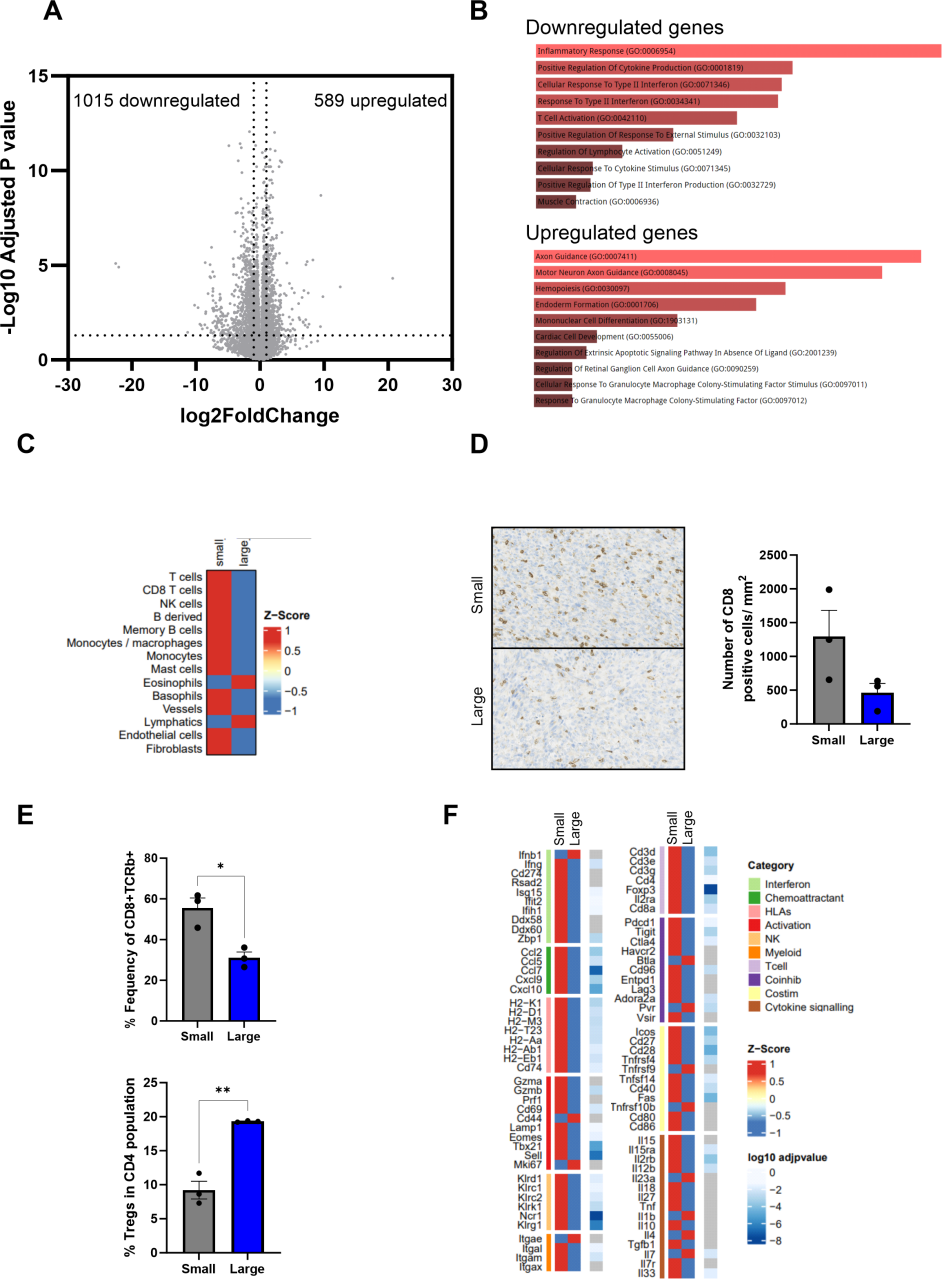
Advanced 4434 tumors are immunologically colder than early disease. 4434 cells (4×10^6^) were injected subcutaneously into C57BL6 mice, tumors were collected when they reached an average volume of either 50 mm^3^ (small) or 150 mm^3^ (large) (n=3 per group). (A) Shows the volcano plot of differentially expressed genes in small versus large tumors. The x-axis and y-axis are the log2(fold change) and log10(p-adjusted) values, respectively (dotted lines indicate twofold change on x-axis and p<0.05 on y-axis). (B) Shows the top-10 differentially enriched GO biological processes (adjusted p<0.05) that are associated with both upregulated and downregulated genes sets. (C) Heatmap showing Z-score normalized mMCP counter scores in small and large tumors. (D) Immunohistochemical analysis of CD8 expression in small-tumors (top) and large-tumors (bottom), quantification of number of positive CD8/mm^2^ was determined by QuPath, mean+SEM is plotted. (E) Tumors were homogenized and cell surface expression and intracellular staining of TCRb, CD8, CD4, CD25 and FoxP3 were analyzed by flow cytometry. (F) Heatmaps showing Z-scores of normalized immune gene expression in small and large tumors. GO, gene ontology; HLA, Human Leuokcyte Antigen; NK, Natural killer; Treg, regulatory T-cells.

While it is well-recognized that immunotherapy is more effective in patients with a lower tumor burden,^17 18^ the evolution of the immune microenvironment as tumors grow, and how this impacts the efficacy of immune-based treatments, is poorly understood. Therefore, prior to testing MG1 in vivo, we first investigated the transcriptional and phenotypical differences between small (50 mm^3^) and large (150 mm^3^) 4434 melanomas growing in C57BL/6 mice. Bulk RNA sequencing was performed on small and large tumors (n=3 mice per group). Differential gene expression revealed that large tumors had 589 genes upregulated and 1015 genes downregulated (by greater than twofold, p<0.05) compared with smaller tumors (figure 3A). To investigate which biological processes were changing in large tumors gene ontology (GO) analysis was performed on both upregulated and downregulated genes. GO processes that were downregulated in large tumors highlighted multiple immunological pathways, such as inflammatory response, T-cell activation and positive regulation of cytokine production (figure 3B). As multiple immune pathways were identified to be dysregulated in larger tumors, we next assessed the total immune and stromal composition of both small and large 4434 tumors using the mMCP counter tool; total T, CD8+, Natural killer (NK), B and myeloid cells were reduced in large tumors compared with small (figure 3C). To validate these changes in the immune composition, the number of CD8+ cells was assessed by immunohistochemistry and flow cytometry analysis. Immunohistochemical and flow cytometry based staining confirmed that within large tumors, CD8+ cells were reduced, both as an individual population (per mm^2^ and as a proportion of total T cells (figure 3D,E, respectively). Moreover, the number of FOXP3+CD4+ cells increased as a proportion of the total CD4+T cell population in larger tumors (figure 3E). To further interrogate the differences in the transcriptomics of small and large tumors, we focused on the expression of a subset of immune-specific genes (figure 3F). Small tumors displayed an increased expression of genes involved in antigen presentation (*H2-K1*, *H2-D1*, *H2-M3*, *H2-T23*, *H2-Aa*, *H2-Eb1*, *Cd74*), co-stimulation (*Icos*, *Cd27*, *Cd28*, *Tnfrsf4*, *Tnfsf14*, *Cd40*) and immune cell recruitment (*Ccl2*, *Ccl5*, *Ccl7*, *Cxcl9* and *Cxcl10*) compared with large tumors. Taken together, small 4434 tumors are immunogenically “hotter” than more advanced larger tumors, which display a reduced immune cell infiltrate and reduced expression in key genes involved in antitumor immune responses.

### T-cell receptor dynamics differ in more advanced tumors

**Figure 4:**
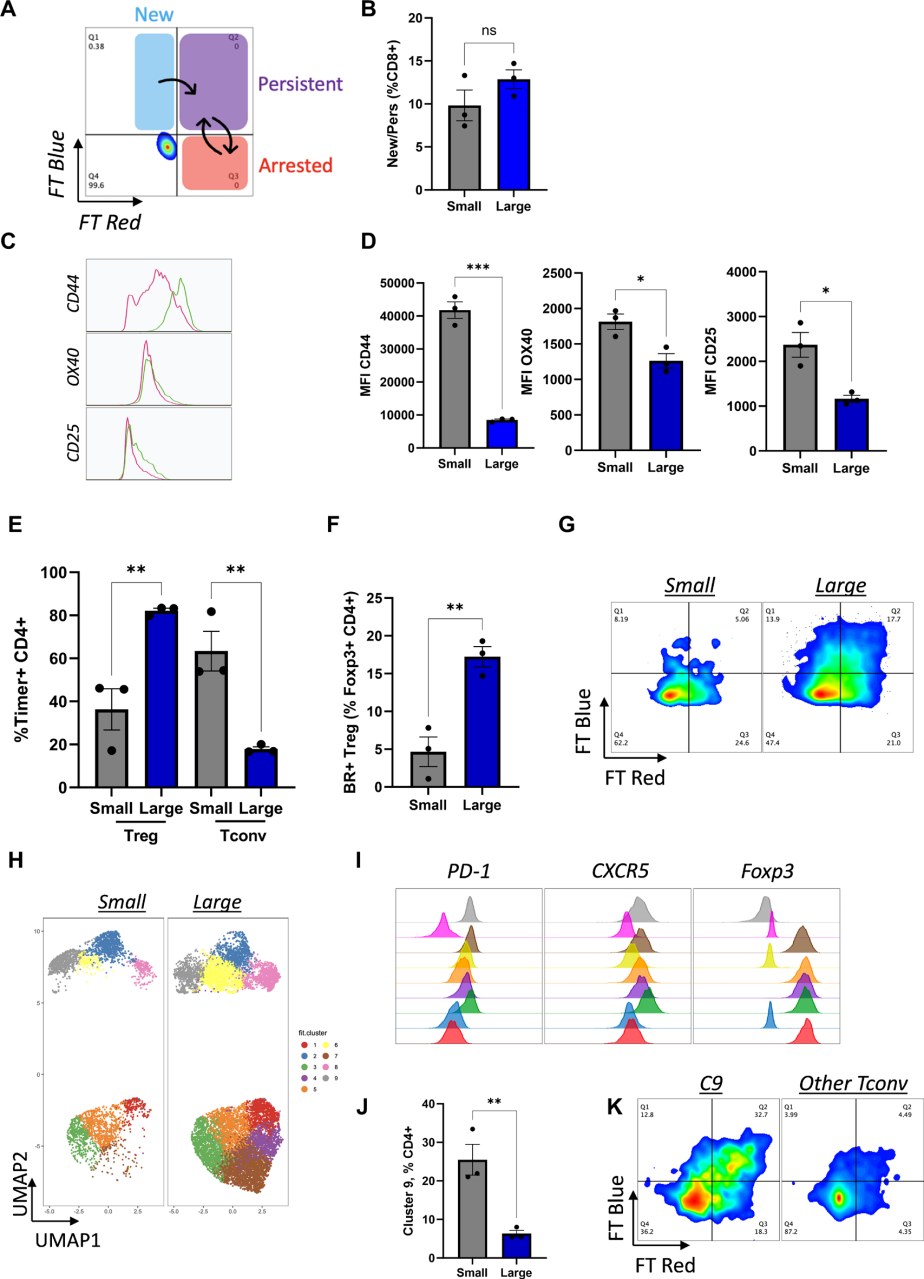
Analysis of the dynamics of antigen engagement in large and small tumors. 4434 cells (4×10^6^) were injected subcutaneously into Nr4a3 Tocky mice, tumors were collected when they reached an average volume of either 50mm^3^ or >100 mm^3^ (n=3 per group) homogenized and analyzed by flow cytometry. (A) Schematic of Tocky fluorescence following antigen engagement, “New” blue+ (B+), “Persistent” Blue+/Red+ (BR+) and “Arrested” Red+ (R+). (B) The percentage of recently engaged (B+/BR+) CD8+T cells. Expression of CD44, OX40 and CD25 on CD8+T cells with recent antigen engagement. (C) Representative histograms (small=green and large=pink) and (D) mean fluorescence intensity values. (E) The percentage of Timer+CD4+ cells in Tconv and Treg T cell subsets. The percentage of BR+Treg in total Treg CD4+FoxP3+ populations (F) representative flow plot shown in G. (H) UMAP cluster analysis of the CD4+TIL population. (I) Percentage of cluster 9 as a percent of CD4+cells. (J) Representative histograms showing expression of PD-1, CXCR5 and FoxP3 on CD4+ TIL clusters. (K) Tocky fluorescence of cluster 9 (C9) compared with other Tconv clusters in small tumors. TIL, tumour infiltrating lymphocytes; Tconv, conventional T cell ;Treg, regulatory T-cells.

A further characteristic of tumors is the dynamics of their TCR signaling, which we have previously shown to impact on oncolytic virus therapy.^19^ Therefore, to further evaluate differences in T-cell function between small and large tumors, tumors from 4434-bearing Nr4a3-Tocky mice that exhibited either limited (<50 mm^3^, small) or enhanced growth (>100 mm^3^, large) at 21 days post-implantation, were analyzed by flow cytometry. The Tocky model is a transgenic mouse in vivo system that incorporates an unstable fluorescent reporter protein in the promoter region for Nr4a3, an intermediate-early gene downstream of TCR signalling.^20^ On TCR signaling, Nr4a3 is transcribed resulting in blue fluorescence, which decays over time to red with a half-life of 6 hours. If the TCR is persistently engaged, and Nr4a3 continually transcribed, new blue fluorescence is seen within the cell in addition to decaying red, resulting in blue/red positive “persistent” T-cells (figure 4A shows an illustrative flow cytometry schematic).^20^ This model enables isolation and analysis of the antigen-reactive T-cell population within a tumor in vivo with a temporal component*,* and in this context provides valuable information regarding the occupancy of the “antigen niche” within the TME as growing tumors escape immune control.

When comparing CD8+T cells within small and large tumors, although no change in the absolute frequency of recently antigen-engaged cells (Tocky Timer Blue+/BlueRed+) was demonstrated between small and large tumors (figure 4B), TCR-engaged CD8+T cells within small tumors had significantly higher expression of the T-cell activation/memory markers CD44 and CD25, and the co-stimulatory receptor OX40 (figure 4C,D), suggesting a better CD8+T cell fitness response to antigen-engagement.

Within the CD4+ compartment, a shift was seen in the composition of the total antigen-reactive population (Tocky Timer+CD4+) between small and large tumors. In large tumors, this population was primarily composed of regulatory T-cells (CD4+FoxP3+ Treg) (figure 4E), which had high levels of persistent antigen-engagement (figure 4F,G), a characteristic of effector Treg.^20^ In contrast, CD4+FoxP3− conventional CD4 T cells (Tconv) predominated within the antigen-reactive CD4+population in small tumors (figure 4E), suggesting a more supportive, less immunosuppressive environment for therapy in smaller tumors. Uniform Manifold Approximation and Projection (UMAP) cluster analysis of tumor-infiltrating CD4+T cells further illustrates this shift (figure 4H) and demonstrates a population of CD4+Foxp3− Tconv that are significantly enriched in small tumors (cluster 9, figure 4H,J).^21^ Marker analysis of this cluster reveals high expression of PD-1 and CXCR5 (figure 4I), characteristic of follicular helper T cells (Tfh). This cluster is highly antigen-engaged when compared with the other Tconv clusters (figure 4K), suggesting Tfh may be instrumental in maintaining an immune response within small tumors. This subset has also been demonstrated to aid response to therapies targeting the PD-1/ programmed death-ligand 1 (PD-L1) axis, although confirmation of their role in cancer remains to be elucidated.^22 23^

### A single intratumoral injection of MG-1 is highly successful in curing early disease burden but is ineffective in advanced disease

**Figure 5:**
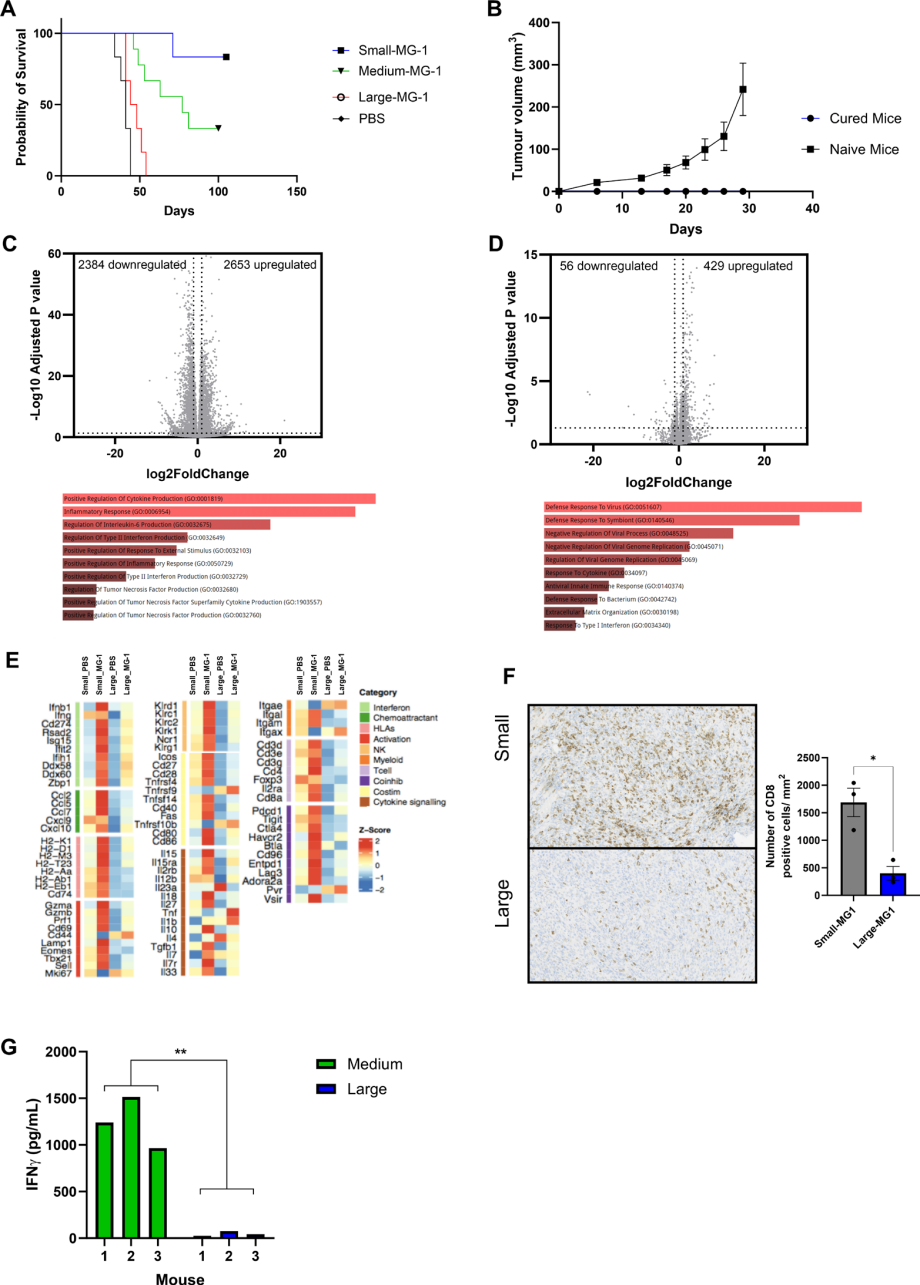
A single intratumoral injection of MG-1 is highly successful in curing early disease burden but is ineffective in advance disease. 4434 cells (4×10^6^) were injected subcutaneously into C57BL6 mice intratumoral injection of MG-1 (1×10^7^ PFU) or PBS were performed on small, medium, and large tumors (50–150 mm^3^). (A) Kaplan-Meier survival curve of tumor bearing mice (six mice per group). (B) Mice cured with MG1 treatment (five mice) were injected with 4434 cells (4×10^6^) alongside naïve mice (six mice) and growth of subcutaneous tumors plotted over time. Graph shows the average tumor growth±SEM. (C and D) Show the volcano plot of differently expressed genes following MG1 treatment of small (C) and large (D) tumors. The x-axis and y-axis are the log2 (fold change) and -log10(p-adjusted) values, respectively (dotted lines indicate twofold change on x-axis and p<0.05 on y-axis). Top-10 differentially enriched GO biological processes (adjusted p<0.05) that are associated with both upregulated and downregulated genes are displayed underneath. (E) Heatmaps showing Z-scores of normalized immune gene expression in small and large tumors±MG1 treatment. (F) Immunohistochemical analysis of CD8 expression in small (top) and large (bottom) 4434 tumors 48 hours following MG1 treatment, quantification of number of positive CD8/mm^2^ was determined by QuPath, mean+SEM is plotted. (G) Splenocytes from individual C57BL/6 mice bearing medium or large subcutaneous 4434 tumors treated with MG1, were re-stimulated in vitro with 4434 tumor cells. 48 hours later, supernatants were assayed for secretion of IFN-γ by ELISA. Graphs show the concentration of IFN-γ from individual mice (three mice/group). HLA, Human Leukocyte Antigen; IFN, Interferon; NK, Natural killer; PBS, Phosphate buffer saline.

With the differences in the TIME observed in early and late 4434 disease states, we decided to test how effective the same dose of MG1 was in tumors that were small (50 mm^3^), medium (100 mm^3^), or large (150 mm^3^). MG1 was highly effective at treating small tumors, curing 90% of animals (figure 5A). Medium tumor-bearing mice were less responsive to MG1 treatment, with 40% of mice displaying long-term survival; the median survival of this group was 77 days compared with 41 days in untreated mice (p<0.0001) (figure 5A). However, MG1-treated large tumor-bearing mice only showed a small increase in survival when compared with untreated mice phosphate buffer saline (PBS) median survival 41 days vs 46 days for large tumor MG1-treated tumors p=0.0486) (figure 5A). To determine whether cured mice generated long-term antitumor immunity, successfully treated, cured mice were re-challenged with 4434 tumors alongside naïve control mice (figure 5B). While naïve mice developed 4434 tumors, no tumor growth was observed in the cured mice, indicating that long-term immunity had been generated following MG-1 treatment, regardless of the initial size of the tumor (small or medium). To understand the global immune effects of MG1 on small and large tumors, we used RNA sequencing to investigate the transcriptional changes occurring within the different sized tumor 48 hours following viral injection. Following MG1 treatment, small tumors demonstrated 5037 significantly differentially expressed genes, while large tumors had only 485, indicating a greater impact on global gene expression in small tumors following MG1 (figure 5C,D, respectively). The GO biological processes that were associated with upregulated genes in small tumors following MG1 treatment, included positive regulation of cytokine production, inflammatory response, and regulation of type II IFN and TNF. Conversely, upregulated genes in large tumors treated with MG1 did not contain any of these processes; instead GO biological processes against virus infection predominated, including defense to virus, negative regulation to virus process and replication, and antiviral innate immune response. Next, we investigated the response to MG1 treatment on our targeted subset of immune-specific genes. MG1- treated small tumors had increased expression of many genes associated with immune activation compared with untreated small, and MG1-treated large tumors, including antigen presentation, chemoattraction, IFN response and cytokine signaling, as well as expression patterns associated with greater immune cell infiltration and upregulation of costimulatory and coinhibitory molecules (figure 5E). CD8 expression in small and large tumors following MG1 treatment was validated at the protein level by immunohistochemical staining; MG1-small-treated tumors demonstrated a significantly higher number of CD8 cells/mm^2^ when compared with MG1-large-treated tumors (figure 5F). Finally, to measure the level of antitumor immunity generated following MG1 treatment, splenocytes were collected from medium or large MG1-treated tumor-bearing animals. Splenocytes from medium-MG1-treated animals displayed a recall response against 4434 tumor cells ex vivo as measured by IFNγ release, while splenocytes isolated from large-MG1-treated animals showed no such responses (figure 5G). These results indicate that the magnitude of the immunological response to MG1, and the generation of tumor-specific T cells is impeded in locally advanced, relative to earlier, disease, due to the more immunosuppressed TIME.

### Combination of MG-1 with αPD-1 improves antitumor immunity and survival compared with monotherapy in locally advanced disease

**Figure 6:**
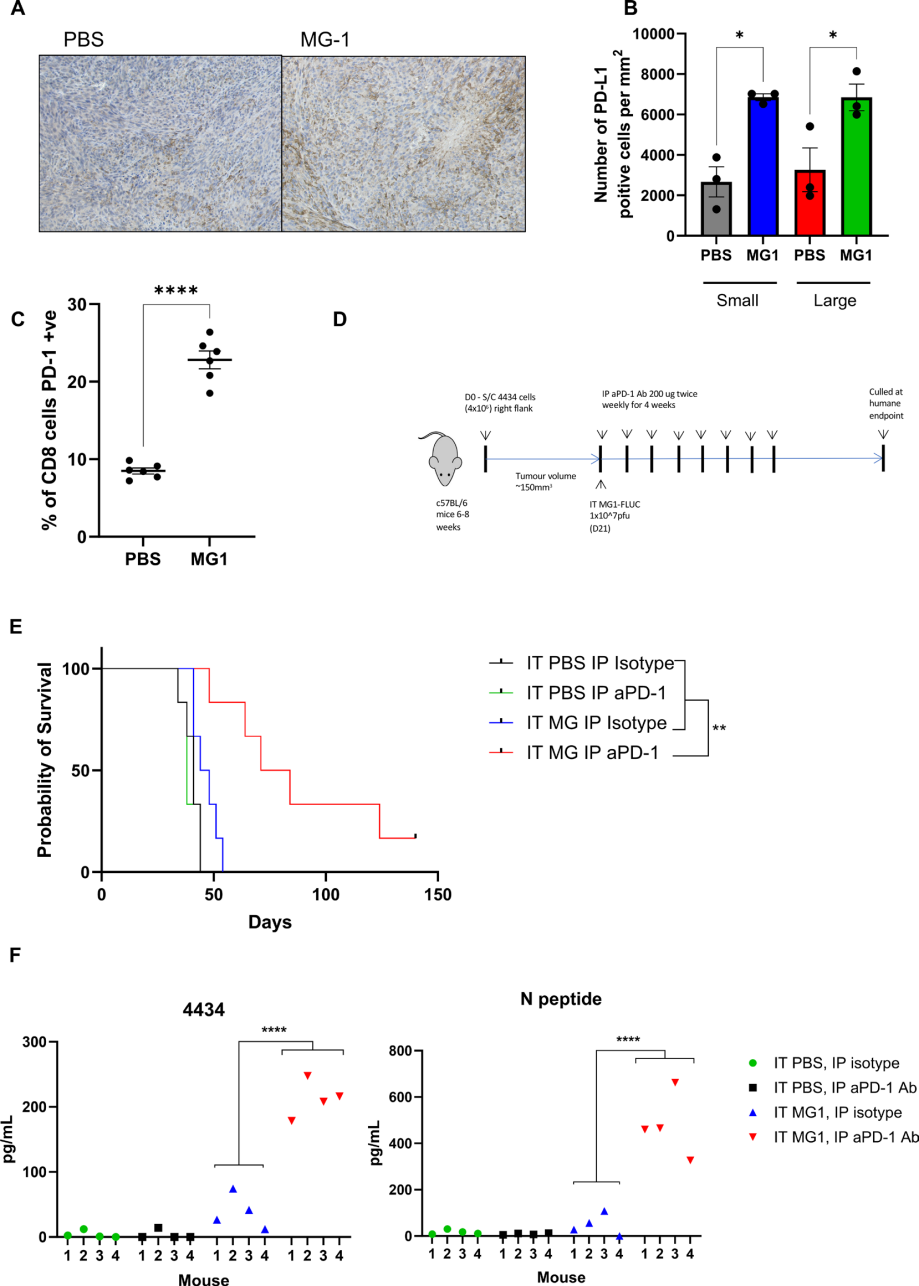
Combination therapy of MG-1 with αPD-1 overcomes resistance of monotherapies in larger tumors. (A) PD-L1 expression was determined by immunohistochemistry in small and large tumors following MG1 treatment (left, large-PBS treated; right large-MG1 treated). (B) The number of positive PD-L1/mm^2^ was determined by QuPath, mean±SEM is plotted. (C) Splenocytes were isolated from PBS (control) or MG-FLUC treated animals and PD-1 expression on CD8 T cells determined by flow cytometry. The mean percentage PD-1 expression on CD8 positive splenocytes is shown±SEM (five mice per group). (D) Schematic of treatment regime. (E) Kaplan-Meier survival curve of treated animals (six mice/group). (F) Splenocytes from individual C57BL/6 mice bearing subcutaneous 4434 tumors and treated with a combination of intratumoral PBS or MG-1 and intraperitoneal isotype control antibody or anti-PD-1 antibody as labeled, were re-stimulated in vitro with 4434 tumor cells or N peptide. 48 hours later, supernatants were assayed for secretion of IFN-γ by ELISA. Graphs shown the concentration of IFN-γ from individual mice (four mice/ group). HLA, Human Leukocyte Antigen; IFN, Interferon; IP, intraperitoneal; IT, intratumoral; MG-FLUC, MG-1 expressing firefly luciferase; NK, Natural killer; PBS; Phosphate buffer saline; PD-1, programmed cell death protein-1; PD-L1, Programmed Death-Ligand 1; S/C, subcutaneous.

As MG1 as a monotherapy against more advanced disease did not generate long-term cures, we hypothesized that the addition of αPD-1 to the treatment regimen would improve the generation and function of antitumor T cells. Importantly, PD-L1 (*Cd274*) and PD-1 (*Pdcd1*) were both upregulated following MG1 treatment (figure 5E). This was confirmed at the protein level by immunohistochemical and flow cytometry-based methods, which demonstrated a significantly increased expression of PD-L1 following MG1 treatment of both small and large tumors (figure 6A,B). Furthermore, PD-1 expression was significantly increased on splenic CD8 T cells 7 days following intratumoral MG1 (figure 6C), indicating a systemic effect of local MG1 administration and thus implicating the PD-1/PD-L1 axis as a valid target for combination immunotherapy. Large 4434 tumors were treated with either a single agent alone (in combination with PBS or isotype control) or MG1 and αPD-1 co-treatment. ICI was given twice weekly following a single MG1 intratumoral injection (figure 6D). MG1 and αPD-1 were ineffective when delivered as monotherapies in this advanced disease setting (median survival PBS/isotype; 41 days, PBS/αPD-1; 38 days, MG1/isotype; 46 days); however, the addition of αPD-1 to MG1 significantly increased survival (median survival 77.5 days) (figure 6E). Ex vivo splenocyte recall assays were performed to assess the level of antitumor immunity generated following combination therapy, the addition of αPD-1 to MG1 treatment significantly enhanced priming against 4434 tumor cells (figure 6F). In this experiment we also tested priming against MG1, by pulsing splenocytes with a defined H-2b restricted rhabdovirus N protein peptide; consistent with the human data from figure 2E, activation of an immune response against tumor was accompanied by an antiviral response, with both responses enhanced in vivo by the addition of αPD-1 to virotherapy (figure 6F). Taken together, these results suggest that MG1 in combination with αPD-1 may be an effective treatment option particularly for more advanced melanoma, with partial reversal of the immunosuppressive microenvironment in larger tumors being boosted by the addition of ICI to reveal a therapeutic effect.

## Discussion

OVs are a promising cancer immunotherapy agent due to their direct lytic effect followed by the generation of antitumor T-cell responses. The induction of an effective antitumor T-cell response is critical to generating long-term responses following oncolytic virotherapy. Therefore, gaining a greater understanding of conditions required for effective T-cell clearance of tumors and the induction of antigen-specific T-cell responses is essential to identify patients with cancer that have the best chance of responding to oncolytic virotherapy, and to facilitate the design of combination therapy strategies. The evolution of the TIME as a tumor grows, and how this impacts the efficacy of immune-based treatments, is poorly understood, and has not been extensively investigated in murine models. Therefore, in this study, we characterized 4434 tumors at different levels of disease burden to try and understand the impact of tumor growth on immune features in the TME and investigated how these changes may impact successful virotherapy. RNA sequencing revealed a significant change in immune composition during tumor growth, which was confirmed at the protein level with immunohistochemical staining and flow cytometry. We found that, as these tumors grew, they became immunological colder with fewer immune cells and a reduction in immune stimulatory genes; while such data may be unsurprising, it provides clear leads on what may be important targets to pursue in the more advanced disease setting. This preclinical data supports the clinical findings that large tumors are more immunosuppressed compared with small tumors,^13 17^ directly impacting the ability of the host immune system to effectively mount a natural or immunotherapy-induced immune response. Although our study focused on the immunosuppressive features of the local TIME, this does not reflect the full nature of the immunosuppressive impact of increased tumor burden, which will be systemic as well as local.^12 24^ Performing similar characterization in other models and correlating these findings with human datasets will be important to validate shared targets of most relevance for testing in mouse models. Interestingly, while both αPD-1 therapy and MG1 were ineffective as single agents in advanced disease, combination treatment led to a significant extension of survival that was associated with an increase in both antitumor and antiviral T cells. It is noteworthy that both our human and mouse systems show priming against tumor is accompanied by priming against virus; however, the importance and the relative contribution of the T-cell response against the virus relative to the tumor for effective oncolytic virotherapy therapy remains unknown. The findings of the current study support the potential importance of the antiviral response in initiating and/or maintaining an antitumor effect and highlight the relevance of tracking both these targets of immune priming as translational readouts within oncolytic virus trials.

Overall, this study shows that combining MG1 with αPD-1 therapy has the potential to overcome therapy resistance in an immunological “colder” advanced tumor TME. Its broader implications highlight both the need to understand the biology of more advanced relative to earlier stage cancer at baseline, so that appropriate treatment targets can be selectively identified and pursued using single agent or combination strategies, and the value of using orthogonal human and murine preclinical systems to maximize the impact of laboratory studies on the design and interpretation of clinical studies. Here, using these approaches, we have rationalized a single-agent OV approach for the treatment of early melanoma, with immune checkpoint combination suitable and required only for more advanced disease. Finally, these findings throw light on negative immunotherapy trials in which the wrong stage of disease may have been targeted.^2^ For example, when αPD-1 was added to oncolytic virotherapy in melanoma, the trial was designed to exclude those patients with the most advanced disease, who had not benefitted from single-agent virus in a previous trial.^25^ Our data suggests that the most advanced patients are those for whom the combination would nevertheless have been of greatest benefit, which may explain why no significant difference was seen with αPD-1±OV in the study as designed.^26^ Hence understanding and incorporation of disease stage into immunotherapy trials in preclinical models may have significant implications for studies as designed and delivered in the clinical setting.

## Material and methods

### Cell culture and reagents

A375, MeWo, T2 and Vero cell lines were purchased from American Type Culture Collection (ATCC) and authenticated using Short Tandem Repeat (STR) profiling and comparison with the The Leibniz Institute DSMZ- German Collection of Microorganisms and Cell Culture database. Mel-624 and Mel-888 were obtained from the Cancer Research UK cell bank. The BRAF-mutant (BRAFV600E) mouse melanoma cell line 4434 was established from C57BL/6_BRAF +/LSL-BRAFV600E; Tyr:CreERT2+/o.^27^ All cell lines were grown in glutamine-containing Dulbecco's Modified Eagle Medium (DMEM) (Sigma-Aldrich), supplemented with 10% Fetal Calf Serum (FCS) (*v*/*v*) (Sigma-Aldrich), apart from T2 cells which were grown in glutamine containing Roswell Park Memorial Institute (RPMI) (Sigma-Aldrich), supplemented with 10% FCS (*v*/*v*). All cell lines were routinely checked for mycoplasma and were free from contamination.

Human peripheral blood mononuclear cells (PBMCs) were isolated and cultured from healthy donor volunteers after written, informed consent was obtained. PBMC were isolated from whole blood by density gradient centrifugation on Lymphoprep (StemCell Technology) and cultured at 2×10^6^ cells/mL in glutamine containing RPMI, supplemented with 10% FCS (*v*/*v*). CD14^+^ cells were isolated from PBMC using Magnetic-activated cell sorting (MACS) isolation procedures, following the manufacturers’ protocols (Miltenyi Biotec). Immature DC (iDC) were generated by culturing CD14+ cells in glutamine-containing RPMI supplemented with 10% FCS (*v/v*), recombinant human IL-4 500 IU/mL and Granulocyte-macrophage colony stimulating factor (GM-CSF) 800 IU/mL (both R&D systems) at a cell density of 1–2×10^6^ cells/mL for 5 days. CTLs were cultured at 4–6×10^6^ cells/mL in in glutamine-containing RPMI supplemented with 7.5% (*v/v*) human AB serum, 1 mM sodium pyruvate, 1 mM 2-(4-(2-hydroxyethyl)-1-piperazinyl)-ethanesulfonic acid (HEPES); 1% (*v/v*) non-essential amino acids, 20 µM 2β-mercaptoethanol (all Sigma-Aldrich) and recombinant human IL-7 (5 ng/mL) (R&D Systems).

### Viruses

MG-GFP and firefly luciferase (MG-FLUC) were provided by Ottawa Hospital Research Institute and virus amplified and titer determined by standard plaque assay on Vero cells.^3^

### MTT cell viability

Melanoma cell lines were treated with MG-GFP at indicated doses for 24 and 48 hours. 20 µL (3-(4,5-dimethylthiazolyl-2)-2,5-diphenltetrazolium bromide (MTT)) (5 mg/mL; Sigma-Aldrich) was added to cells 4 hours prior to the end of the incubation period. After 4 hours, tissue culture supernatant was removed, and cells were solubilized using 150 µl Dimethylsulfoxide (DMSO) (Sigma-Aldrich). Optical density absorbance readings were determined using a Thermo Multiskan EX plate reader (Thermo Fisher Scientific), at 540 nm absorbance.

### Infection of tumor spheroids

Mel-888, MeWo and Mel-624 cells were seeded into 96-well ultra-low binding plates (Corning) at a density of 2.5×10^4^ cells/well and cultured for 5 days. Spheroids were infected with MG-GFP at MOI 0.1, GFP images were taken 20 hours post infection and fluorescence measured by the Cytation 5 Imaging Plate Reader (BioTek). Viral replication was measured from supernatants collected 24 hours post infection and viability determined at 48 hours by 3D CellTiter-Glo assay following the manufacturer’s instructions (Promega).

### Enzyme-linked immunosorbent assay

The production of human IFNα (Mabtech), IL-28, IL-29, IP-10 (R&D Systems), IL-10 and TNFα (BD Biosciences) and murine IFNγ (R&D Systems) in cell-free supernatant was determined using matched-paired antibodies according to the manufacturer’s instructions. Optical density absorbance readings were determined using a Thermo Multiskan EX plate reader, at 405 nm absorbance.

### T2 stability assay

T2 cells were seeded at 1×10^6^/mL with 10 ug/mL MG-1 peptide’s (TLLKATPA, RLGPTPPML, SLIQDVERI, GLSKNPVEL, QMTPDVYKV, VLNAPPVQM, KLMDGLINQC, YLYAALKEI, YLLGLYRV; all JPT) cultured for 10 mins at 37°C, then incubated at room temperature overnight. T2 cells were then cultured at 37°C for 2 hours. T2 cells were then cell surfaced stained for HLA-A2-PB450 or isotype control.

### Cell surface phenotyping

Cell surface expression of indicated markers were quantified by flow cytometry. Briefly, cells were harvested, washed in FACS buffer (PBS; 1% (v/v) FCS; 0.1% (w/v) sodium azide), incubated for 30 mins at 4 °C with specific antibodies or matching isotype controls. Cells were wash with FACS buffer and then fixed with 1% Paraformaldehyde (PFA) (1% (w/v) paraformaldhyde in PBS) and stored at 4 °C prior to acquisition. Flow cytometry analysis was performed either using BD LSR II flow cytometer Cytek Aurora Spectral cytometer or CytoFLEX S and analysis carried out using either FlowJo software V.0.9 or CytExpert software V.4.3.

### Intra-cellular staining

Cells were cell-surfaced stained and fixed overnight with 1% PFA prior to permeabilization with 0.3% Saponin (Sigma-Aldrich) for 15 mins at 4 °C. Cells were wash with 0.1% Saponin, incubated with specific antibodies or matched isotype controls for 30 mins at 4 °C. Cells were wash with PBS and flow cytometry analysis was performed immediately using the CytoFLEX S.

### Flow cytometry antibodies

Human: CD11c APC-Vio770 (MJ4-27G12, Miltenyi Biotec), CD14-PerCP (TUK4, Miltenyi Biotec) CD86-PE-Cy7 (2331, BD Biosciences), CD80-PE (L307.4, BD Biosciences) HLA-ABC-VioBlue (REA230, Miltenyi Biotec), HLA-DR/DP/DQ-FITC (Tu39, BD Biosciences), CD3-PerCP (SK7, BD Biosciences), CD8-APC (RPA-T8, BD Biosciences) IFNg-BV421 (4S.B3, BD Biosciences), HLA-A2-PB450 (BB7.2). Mouse IgG1, κ Isotype Control (PE/FITC/PerCP/PE-Cy7/APC) (MOPC-21, BD Biosciences), Mouse IgG2a, κ Isotype Control (FITC/PE) (G155-178, BD Biosciences), REA Control-VioBlue (REA293, Miltenyi Biotec) and Mouse IgG2bk-PB450 (MPC-11). Mouse: CD3 Per-CP (17A2), CD4 BUV395 (GK1.5 BD Biosciences), CD8a BV785 (53–6.7 BD Biosciences), CD8a BV650 (53–6.7), TCRb BUV737(H57-597 BD Biosciences),FOXP3-AF488 (MF23), CD25 AF700 (PC61) PD-1 PE-Cy7 (29F.1A12), PD-1-APC (JK3) CXCR5-PeCy7 (DPRCL5) OX40-BV711 (OX-86), all purchased from BioLegend unless stated otherwise.

### Cytotoxic T-cell priming assay

Tumor spheroids were infected with MG1 at a MOI 0.1, then cultured with iDC at a 3:1 tumor:iDC ratio for 24 hours. iDC were collected, washed in PBS and then cultured with autologous PBMC at a 30–40:1 PBMC:iDC ratio for 7 days. Where applicable RNA was collected from week 1 CTL then re-stimulated and cultured for a further 7 days. Primed CTL (week 2) were then harvested, RNA collected, used peptide recall assay or ^51^CR killing assay.

### Peptide recall assay

To measure peptide-specific CTL responses autologous CD14+ cells were incubated with either premelanosome (PMEL), Tyrosinase (TYR) or melanoma antigen recognized by T-cells 1(MLANA) PepTivator peptide pools (15-mer peptide sequences with 11 amino acids overlap, Miltenyi Biotec) for 60 min at 37°C, according to the manufacturer’s instructions. Autologous CD14+ cells loaded with specific, or control peptide were then co-cultured with CTL for 60 min at 37°C, Brefeldin A (1:1000, BioLegend) and CD8-APC were then added to cultures and incubated for a further 4 hours at 37 °C. Cells were fixed and then intracellular IFNγ-BV421 staining performed prior to analysis by flow cytometry.

### ^51^Cr release assay

Week 2 CTL were cultured with 10 ug peptide-loaded T2 cells (or unloaded control) at 50:1 Effector:Target (E:T) ratio for 4 hours (cells were then pelleted by centrifugation and 50 µl of supernatant was transferred to scintillation plates (Perkin-Elmer)) prior to analysis using a Wallac Jet 1459 Microbeta scintillation counter and Microbeta Windows software (Perkin-Elmer). Percentage lysis was determined using the following calculation:

% lysis= (Sample CPM − Spontaneous CPM) / (Maximum CPM − Spontaneous CPM) × 100

### TCR sequencing

PBMCs from two different donors (D1 and D2) treated under two different conditions (mock and MG1) and two time points (week-1 and week-2) were used for TCR sequencing. The sequencing was performed in triplicates. RNA was extracted using TriZol following manufacturer’s instruction (Invitrogen). RNA quantification and quality analysis was performed using Qubit Fluorometry HS kit (Thermo Fisher Scientific) and TapeStation (Thermo Fisher Scientific), respectively, according to manufacturer’s instructions. Bulk RNA-based sequencing of the CDR3β chain was done using a multiplex PCR protocol as previously described.^28^ Starting material was 500 ng of RNA diluted in 8 uL of RNase-free water. Briefly, this method is multiplex PCR-based and uses 38 primers against the TCR V genes, incorporating unique molecular identifiers to allow the quantification of TCR clones and for the correction of amplification biases and sequencing errors. Pooled libraries were sequenced according to the Illumina MiniSeq protocol in high output mode (2×150 bp paired-end), typically yielding a coverage of 150 K reads per sample. The CDR3β extraction and quantification was performed using a computational pipeline for TCR analysis available as a suite of Python scripts available at https://github.com/innate2adaptive/Decombinator.

For the analysis, the TCR repertoires from the triplicates were merged based on donor and condition. Week-2 clonotypes were then classified according to their normalized expansion rate. The expansion rate for each TCR was calculated based on the formula:

$$Expansion\;rate=\;\frac{\frac{frequency\;at\;week-2\;}{singleton\;frenquency\;at\;week-2\;}}{\frac{frquency\;at\;week-1}{singleton\;at\;week-1}}$$

TCRs were classified as expanded if normalized expansion rate was >1 or contracted if normalized expansion rate was <1. Each repertoire group was subsequently clustered based on CDR3β amino acid triplet similarity using a kernel matrix and a similarity threshold of 0.65. Normalized cluster count (to the top 500 TCRs) and cluster size were quantified.

### Animals

5–6 weeks old female C57Bl/6 mice (Charles River, UK) or Nr4a3 Tocky mice (C57Bl/6 background) were used for tumor implantation. These were conducted at the ICR Biological Services Unit and were approved by the ICR local Ethical Review Committee (Project license: PP7490522) and standards of care were based on the United Kindom Co-ordinating Committee on Cancer Research (UKCCCR) Guidelines for the welfare and use of animals in cancer research. 4434 murine melanoma tumors were established by 100 µL subcutaneous injection of 4×10^6^ cells respectively into the right flank of each mouse. Tumor measurements were taken twice weekly in three dimensions using Venier calipers and the tumor volume estimated using the formula: length × width × height (mm) × 0.5236. All animals that grew 4434 tumors were included in treatment groups, and there are no exclusions in analysis. Mice were allocated to groups based on tumor size, endpoint for experiments were predefined or when the tumors reached 1500 mm^3^ limit. For flow cytometry analysis, tumors were harvested at indicated volumes and were mechanically digested with scissors, then digested in 1 mL of a digestion mix containing 25 ug/mL Liberase, 250 uL/mL DNASEI, 40 uL/mL Trypsin at 37°C for 10 mins, then room temperature for 20 mins. Digested contents passed through 70 uM cell strainer and washed with FACS buffer.

### RNA sequencing of 4434 tumors

4434 tumors were explanted from animals 48 hours following treatment and stored in RNALater (AM7020, Thermo Fisher) at −20°C prior to RNA extraction. Samples were homogenized as described previous and RNA extraction performed using RNEasy kit (74104, Qiagen, Germantown, USA) as per manufacturer protocol.

### Bioinformatics analysis

Trimmomatic (V.0.39) was used for raw reads trimming, followed by Hisat2 (V.2.1.0) alignment software for trimmed reads mapping to Ensemble GRCm38.102 mouse reference genome. Stringtie (V.2.1.4) in combination with custom python script were used to generate gene count matrix for all samples. Genes that had less than one read per sample were removed from further analysis. Differential expression analysis was performed in R using the Bioconductor package DESeq2 and mMCP counter was applied to estimate immune and stromal cell type signatures for each sample. Over-representation analysis of differentially expressed genes was carried out in using enrichR web-application.

### Virus treatments

A single intratumoral dose of PBS or MG-FLUC (1×10^7^ PFU) was given when tumors reached the desired volume. For combination experiments 200 ug of isotype control (InVivoMab mouse IgG2b isotype control, clone MPC-11; 2BScientific), or anti-PD-1 (InVivoMab rat anti-mouse PD-1 (CD279), clone- RMP1-14 monoclonal antibody, IgG2a κ; 2BScientific) antibody was administered intraperitoneally two times a week for a maximum of 4 weeks or until mice reached humane end point. Tumor measurements were recorded twice weekly.

### Histology and immunohistochemistry

Tumors from treated mice were dissected and fixed overnight in formalin. These were then processed, embedded and 2 µm sections were prepared on APEX glass slides. Sections from formalin-fixed paraffin-wax embedded tumors were stained with primary rat anti-mouse monoclonal anti-CD8 antibody (eBioscience, 4Sm15as) and goat anti-mouse polyclonal anti-PD-L1 antibody (R&D systems, AF1019). Slides were scanned and imaged using Hamamatsu Nanozoomer (Hamamatsu Photonics).

### Splenocyte recall assay

Spleens were immediately excised from euthanized mice and dissociated in vitro to achieve single-cell suspensions. Red blood cells were lysed with Ammonium-Chloride-Potassium (ACK) lysis buffer for 1 min. Cells were resuspended at 1×10^6^ in glutamine containing RPMI, supplemented with 10% FCS and 1% Pen-Strep. Splenocytes were cultured either alone or with the indicated tumor cells at a ratio of 5:1 (E:T) or Rhabdovirus N peptide (RGYVYQGL). Cell-free supernatants were collected 48 hours later and tested by IFNγ ELISA.

### Statistical significance

Statistical analysis was carried out with the GraphPad Prism software. Statistical differences among groups were determined using student’s t-test, one-way analysis of variance (ANOVA) or two-way ANOVA analysis. For survival experiments, the Kaplan-Meier survival curves were compared using log-rank (Mantel-Cox) test. Statistical significance was determined as follows: *p<0.05, **p<0.0021, ***p<0.0002 and ****p<0.0001.

## Data Availability

Data are available in a public, open access repository.
